# The rediscovery of Palmyra and its dissemination in *Philosophical Transactions*

**DOI:** 10.1098/rsnr.2015.0059

**Published:** 2016-03-16

**Authors:** Gregorio Astengo

**Affiliations:** The Bartlett School of Architecture, University College London, Gower Street, London WC1E 6BT, UK

**Keywords:** Palmyra, antiquarianism, travellers' accounts

## Abstract

This paper examines the first publicly documented western encounter with the ancient city of Palmyra as an archaeological site. This encounter was achieved in the late seventeenth century by a group of British merchants, who reached Palmyra and made drawings and reports of its ruins. The reports were then published in *Philosophical Transactions* in the mid 1690s. This paper points to the ways in which such accounts came into being, as well as how the city was described and publicly communicated for the first time in *Philosophical Transactions*. These articles had a great impact throughout the following centuries as a reference for the study of Palmyra. This paper therefore also stresses the pivotal role of *Philosophical Transactions* for the production and dissemination of Palmyra's archaeological legacy, as well as for the development of early modern archaeology within the early Royal Society.

## Introduction

In the morning of 4 October 1691 a group of 30 men arrived at the ancient city of Palmyra, in the middle of the Syrian desert.^1^ They had departed six days earlier, on 29 September, from Aleppo and had proceeded southeast into the dry wilderness, with the aid of servants and a local guide. The company stayed in Palmyra for four days, leaving in the early morning of 8 October. They made their way east and, after reaching the river Euphrates, they proceeded north along the river, getting back to Aleppo on 16 October.

This short journey, which lasted just 18 days, was one of the first documented western encounters with Palmyra (also known as Tadmor in modern Israel) after hundreds of years. More importantly, this was the first publicly documented archaeological journey to the city, made with the sole intention of studying its remains. Accounts of this travel were attentively recorded and were brought back to London a few years later. They were all published in *Philosophical Transactions* in issues 217 and 218, in October, November and December 1695. This paper addresses the production and dissemination of information about the city of Palmyra during the journey of 1691 and the impact of these reports on the community of scholars working in and around the Royal Society. More specifically, the paper attempts to assess the importance of the articles published in *Philosophical Transactions* within the history of early modern scientific communication at the Royal Society. The production of reliable, informative and ultimately useful information was a crucial component of these reports. Stephen Shapin has remarked the pivotal role of issues of credibility and trustworthiness in shaping modern notions of knowledge-making.^2^ In this paper I draw from such issues, especially relating to communication strategies and their impact on the public realm through both texts and illustrations. Both media had a role in the establishment of Palmyra as part of a common legacy. As Shapin suggests, ‘the transformation of mere belief into proper knowledge was considered to consist of the transit from the perceptions and cognitions of the individual to the culture of the collective’.^3^

The rediscovery of Palmyra, and its publication in *Philosophical Transactions*, also fostered original antiquarian studies on its ancient history, architectural ruins and epigraphic remains. Most of these contributions came from the circle of the Royal Society. Contributors included Edmund Halley, Thomas Smith, Edward Bernard, Robert Huntington and other scholars who were part of the productive circle of orientalists that developed at Oxford.^4^ The importance of this body of work will therefore also be assessed within the development of antiquarian practices in and around the early Royal Society. The late seventeenth century saw the rise of antiquarian studies as an identifiable independent practice with its own specific methods. The Royal Society was one of the centres of this change, in which attentive philological analysis made way for first-person fieldwork and material investigations as the primary and most reliable sources of knowledge.^5^ Antiquities, archaeology and ancient architecture were *de facto* part of the early Royal Society's agenda. Although officially rejecting these subjects as external to their interests, meetings at the Royal Society and articles in *Philosophical Transactions* dealt extensively with these topics. As Oldenburg states in the journal in 1671: ‘For this New Philosophy we were disciplined by the laudable examples of the Most Ancient Sages of the Past. And we had the same of the like guides (no less than the same Old Authority) … for military, civil, and naval Architecture.’^6^

*Philosophical Transactions* was indeed an important ground for promoting antiquarian research, as demonstrated by the works of Martin Lister, Ralph Thoresby, John Lyster and the aforementioned Thomas Smith, especially from the 1680s onwards.^7^ The reports on Palmyra therefore also stand as a contribution towards the establishment of antiquarianism and early modern archaeology as specific branches of scholarly culture. *Philosophical Transactions* actively worked in spreading first-hand knowledge about Palmyra to a vast readership, contributing at the same time to its own authorial identity through the diffusion of new research. The journal was attempting to establish proper standards for the collection of data and first-hand information gathered from around the world. Early issues of *Philosophical Transactions* included ‘Enquiries’ on countries such as Turkey, Persia, Hungary, Egypt, Japan and North America. The journal also proposed guidebook-like articles for travellers, with guidelines and suggestions for seamen and explorers.^8^ The importance of travels and travel accounts had already been mentioned by Francis Bacon in his *Novum Organum* as well as in his essay *Of Travel* and was certainly in line with the empirical approach envisioned and conceived at the Royal Society.^9^ This paper will therefore also point to the role of contributions such as those on Palmyra on shaping the scholarly programme of *Philosophical Transactions* itself. In this sense, the continental impact of these reports should be understood as essential to their success. The dissemination of the reports on a European scale—including France, The Netherlands and Italy—demonstrate their international appeal as well as the fertile network of scholarship on which they could expand.

## Exploring the city

The party who made this historical expedition consisted mostly of merchants and traders who were part of the British Levant Company, working in the trading centre of Aleppo.^10^ An attempt to reach the city had already been made in the summer of 1678, when 16 of these English merchants, together with 24 servants, had departed from Aleppo, on 18 July. Among them was Robert Huntington, who was Chaplain of Aleppo from 1670 to 1681.^11^ They approached Palmyra on 23 July, but soon found themselves trapped and threatened by the local prince, the Emir Melkam (or Milheym), who feared they would reveal his position to the Turks. They were forced to pay their way out of the city with almost everything they owned and returned to Aleppo on 29 July, having lost most of their possessions and having collected nearly no information about the city. The second attempt, made 13 years later, was more fortunate. The city was still inhabited by Arabs, but the company was assured of security and was given supplies and assistance by the local king, Assyne.

**Figure 1.**
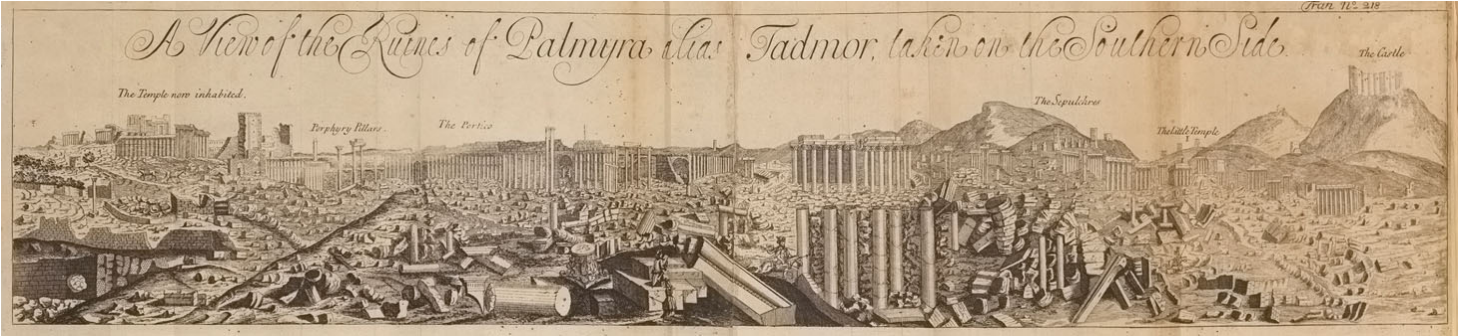
‘A view of the Ruines of Palmyra alias Tadmor, taken on the Southern Side’. (From *Phil. Trans. R. Soc. Lond.* **218**, before p. 125 (1695).) (Online version in colour.)

The reports produced during the first and second journeys were delivered to the Royal Society and consisted of a letter and an extract from two travel journals. The letter contained a description of the city and was written by Mr William Halifax (or Hallifax) during the 1691 expedition.^12^ Halifax graduated from Corpus Christi College in Oxford in 1978 and became a fellow in 1682. Like Huntington, Halifax, too, was a man of the church: he was Chaplain of Aleppo from January 1688 to December 1695, when he resigned and came back to London.^13^ As for the travel journals, they belonged to two Freemen of the Company:^14^ Timothy Lanoy, who had been there since the mid 1670s—his father Benjamin was Consul of Aleppo from 1659 to 1672^15^—and Aaron Goodyear, who had been trading in Aleppo from as early as 1670.^16^ Their travel accounts described both the disastrous journey of 1678, in which they took part with Huntington, and the successful one of 1691. The journals were personally brought back to London by Lanoy and Goodyear and published in the next issue of *Philosophical Transactions.*^17^ This last entry included an engraving nearly 70 cm long, copied from a sketch made on site, depicting a ‘View’ of the city of Palmyra taken from the northeast (figure 1).^18^ This is the first published image of Palmyra and it captures in a single view of almost 180° nearly the whole city. Together with the letter and the travel diaries, the illustration completes this public account on the remains of Palmyra.

In his account, the author, William Halifax, provided a thorough description of the company's visit, following their steps throughout the city. The party, arriving from the north, was immediately welcomed by the view of the late medieval castle of Fakhr-al-Din al-Ma'ani, also known as Palmyra Castle or Tadmor Castle, built during the thirteenth century (Halifax doubtfully proposed 1585 as the date of its completion). The castle stood on a high hill, overlooking the city from the northwest. Described as ‘a Work of more Labour than Art’, the castle clearly did not fulfil the visitors' thirst for classical and ancient architecture, as it was ‘no Old Building’, ‘confused’ and ‘ill contrived’, and did not retain the ‘foot-steps of the exquisite Workman-ship and Ingenuity of the Ancients’.^19^

Having admired the view of the city and its surroundings from the top of the hill, the group started their account from the southeast, more precisely from the Temple of Bel, the greatest and—at the time—the best-preserved construction of Palmyra, built during the first century. As the explorer noted with much disappointment, the Temple had been converted into an Islamic citadel during the twelfth century. Halifax recognized the overlays and additions to the original building, made mostly with elements taken from the ancient temple inside the Temenos. The original Propylaea in front of the main gate were walled with ‘old Stones and many Pillars broken or sawn asunder, being rolled into the Fabrick, and ill cemented’, leaving a narrow entrance in the middle. The western wall was mostly ‘broken down’, and the main gate was ‘a new Building upon an old’.^20^ Entering the Temenos through this gate, the company was amazed by the ‘stateliness’ of the complex. Halifax admired the inner side of the gate, a ‘Magnificent Entrance’ with beautiful stones ‘carved with Vines and Clusters of Grapes, exceeding bold and to the Life’.^21^ The temple itself had been converted into a mosque, with ‘new Ornaments’ and ‘*Arabick* Inscriptions’, not as beautiful as some older ‘Relicks of much greater Artifice and Beauty’ on the northern side of the building.^22^ Inside the Temenos, the merchants also found a few hundred ‘poor, miserable, dirty People’, including the sheik, who lived in ‘little Hutts made of dirt’. The sight surprised and disappointed the visitors, as ‘Certainly the World it self cannot afford the like mixture of Remains of the greatest State and Magnificence, together with the extremity of Filth and Poverty’.^23^ On the northern side of the Temple of Bel they observed ‘a Dome or a Cupola’, which measured ‘above six Foot in Diameter’. This structure was ‘found above to be of one piece’ and Halifax conjectured whether it was ‘hewn out of a Rock entire, or made of some Artificial Cement or Composition, by Time hardened into a Lapideous Substance’.^24^ Halifax was inclined to believe the latter, whereas in fact his first hypothesis was correct. The ceiling of the northern shrine—as well as the southern one—was in fact obtained from a single stone slab.^25^

Once outside this temple, the visit proceeded northwest towards the Monumental Arch, passing by an abandoned mosque and a group of pillars. From the arch, triangular in plan, began the Great Colonnade. While walking along the colonnade, they passed by the Baths of Diocletian, mistaken for a banqueting house.^26^ The group then advanced through this long ‘Piazza’, passing by the theatre, which they mistook for a ‘Royal Palace’.^27^ This was ‘so entirely ruined’ that Halifax could not judge on ‘its Ancient Splendour’.^28^ After having broken off a few slivers of stone to take with them, Halifax briefly mentioned the aqueduct, a ‘Current of hot sulphureous Waters’, which reminded him of the British city of Bath.^29^

The attention of the writer then turned towards the northern part of the city and what he described as an undefined ‘Wood of Marble Pillars’. Halifax was able to recognize a group of 11 pillars forming a rectangular plan, which was probably one of the many wealthy houses that had once occupied the northern side of the city.^30^ They then walked to the Temple of Baal-Shamin, called by them ‘Little Temple’. This ‘very curious’ prostyle tetrastyle temple with a deep porch was completed with both classical and middle-eastern features, such as windows opened on the sides.^31^ The short description of this beautiful building concluded the account of the main city.

The company finally made their way into the Valley of Tombs, the necropolis located on the western side of Palmyra. Here they came across a few dozen square towers, as high as 15 metres, ‘all of the same Form, but of different Splendour and Greatness’. Halifax initially thought them to be fortified bastions or possibly ancient church steeples—in which case they would probably have meant a great deal to the chaplain as old ‘footsteps of Christianity’. Two of these tombs were richly described in the account: they ‘stood almost opposite to one another’ and both had several floors of niches ‘of good Marble’, with ‘very lively carvings and Paintings and Figures’ all around the walls and ceilings.^32^

After having led us ‘up and down’ Palmyra, the company left the city. Halifax briefly narrated their way back to Aleppo, and the travel diary of Lanoy and Goodyear described their state of mind when concluding the survey:

having tired ourselves with roving from Ruine to Ruine, and romaging among old Stones, from which little Knowledge could be obtained … we departed from *Tadmor*, being very well satisfied with what we had seen, and glad to have escaped so dreaded a Place, without any trouble or pretences upon us; but else with some regret, for having left a great many things behind, that deserved a more particular and curious inspection.^33^

## Narrating the city

Once they arrived in London, these accounts of Palmyra were welcomed as extremely valuable pieces of literature. Indeed, collecting and disseminating information on ancient artefacts and historical data was a complex matter. Travellers and readers alike had to deal with several issues both at home and abroad:^34^ from problems relating to the textual and visual description of the sites to questions of authority and credibility of the accounts coming from foreign countries. Palmyra was no exception: these issues, together with difficulties relating to travelling, reporting, measuring, selecting and interpreting ancient and exotic data can all be observed in the accounts made by Lanoy, Goodyear and Halifax. As mentioned above, the company had tried to reach the city years before without success, and the dangers of their expeditions were thoroughly described. Moreover, Halifax's description of Palmyra was far from comprehensive: the author had in fact selected—either by intention or by necessity—a small number of specific buildings, somehow bypassing the whole western part of the city and several other notable ruins.^35^

It should again be stressed at this point that the company was made essentially of merchants and traders.^36^ It is not unlikely that the visitors had an ongoing interest in antiquities and collecting, as this was a common practice in late seventeenth-century Britain.^37^ Journeys such as this were normally motivated by the travellers' own personal interests.^38^ What led Halifax and the company to undertake such a long and dangerous journey was therefore probably a mixture of archaeological fascination and a desire to discover what was considered at the time to be a truly mysterious city.^39^ In particular chaplains, such as Halifax, were often led to carry out their duty abroad by an adventurous interest in the classical world and in ancient antiquities.^40^ Palmyra was uniquely located in the middle of the desert, apparently showing very few natural advantages, but at the same time featuring an incredibly rich and grandiose architecture. Halifax noted this apparent contradiction during his visit: ‘The city of *Tadmor* [is] ill contrived for a place of Trade, being far from the Sea, and without the Advantage of any River. Yet the Magnificence of the Place shews they have not wanted Riches among them.’^41^

Palmyra in fact flourished between the first and third centuries a.d. as an important commercial crossroads.^42^ The city was badly damaged after its famous Queen Zenobia was defeated by the Romans during the second half of the third century, and was later captured by the Muslims. Palmyra passed through the centuries, occasionally experiencing some degree of prosperity, but mostly as an Arab and Ottoman annexed province. The city had always had an interestingly hybrid appearance, more orthodox than other Roman provinces in Syria but of uniquely grand proportions. We can then imagine the company being understandably curious to see this ‘minor Atlantis’^43^ with their own eyes. From what we read, they were mainly interested in the general and social history of the city, its ancient origins, its rulers and possible links with biblical scriptures. In fact, since Pliny's *Naturalis Historia*, Palmyra was generally believed to have been founded by King Solomon, a figure very well known among members of the Royal Society. He was presented by Francis Bacon as the founder of the celebrated Solomon's House, the intellectual institution described in his *New Atlantis* (1627). This institution was probably one of the references for the foundation of the Royal Society.^44^ The discovery of Palmyra could then somehow also contribute to the establishment of the Society's institutional tradition. Halifax and his companions were genuinely impressed by the grandiose size of Palmyra, and when they first set eyes on the remains the author commented:

You have the prospect of such Magnificent Ruines, that if it be Lawful to frame a Conjecture of the Original Beauty of the place, by what is still remaining, I question somewhat whether any City in the World could have challenged Precedence over this in its Glory.^45^

Halifax's account is in fact filled with personal judgements and comments about the beauty of the ruins. Inside the Temple of Bel he noted ‘two Niches for Statues at their full length, with their Pedestals, Borders, Supporters, and Canopies, carved with great Artifice and Curiosity’.^46^ The Temple itself had ‘a most Magnificent Entrance on the West, … which … seems to have been one of the most glorious Structures in the World’.^47^ The Monumental Arch was ‘vastly large and lofty, and for the exquisiteness of the Workmanship not inferiour to any thing before described’.^48^

Halifax also noted more recent constructions alongside his preferred classical buildings. Just outside the Temple of Bel, Halifax noted a mosque that was ‘not worthy to stop us in the way to things both of greater Antiquity, and every way more Noble and worthy our Consideration’.^49^ ‘*Turks*, zealous Enemies of all Imagery’ and ‘Enemies to every thing that is Splendid and Noble’, were often deemed responsible for the ruinous state of the city.^50^ They were accused of destroying the statues that stood on many of Palmyra's monumental columns. As mentioned above, improper use of ancient buildings was also noted. Halifax lamented the inappropriate use of building materials to fortify the Propylaea in the Tempe of Bel, and the ‘hard fate’ of other pieces of porphyry, ‘debased to support the Corner of a little Hutt, scarce enough for a Dog-kennel, or a Hog-sty’.^51^

Halifax's narrative was divided into 10 ‘sections’, which are indicated in the margins of the pages of *Philosophical Transactions*: ‘*Tadmor* Castle’, ‘The Valley of Salt’, ‘*Tadmor*’, ‘The Temple’, ‘A Mosque’, ‘An Obelisk’, ‘The Banquetting-House’, ‘The Palace’, ‘The little Temple’ and ‘The Sepulchres’. These points of interest worked almost as a textual ‘map’. Indeed, the whole account of the city was described through the eyes of the narrator, and is therefore necessarily partial and subjective. The narrative takes us through the streets and buildings of Palmyra, directly addressing the reader with the frequent use of the second person.^52^

This transposition of the reader into the viewer through the text points to the importance of spatial communication and representation strategies. Steven Shapin has suggested that the issue of authority when dealing with late seventeenth-century travellers' tales was often a problem of space. Information had to be efficiently transmitted and transported through space—and often time—without losing factuality and credibility.^53^ The main strategy adopted by Halifax for turning his experience into *matters of fact* was the narrative. Literary communication of such personal perceptions was the most effective way of engaging the public. The description of circumstantial events and details could facilitate what Shapin calls ‘virtual witnessing’, namely the projection on to the reader's mind of such perceptions that actual witnessing could become essentially unnecessary.^54^ Thus the partiality and subjectivity of Halifax's first-person description of Palmyra suggest authorial motives. The intention of such accounts was to reproduce sensory insights acquired by the author and to make them available to the reader. Additionally, interchanging the first person with the second person would put the reader in the travellers' position in both space and time, further enhancing this bodily and visual shift.

These strategies relate closely to the communication of scientific facts and experiments made at the Royal Society in the 1660s and 1670s, which were often written and published as personal reports in *Philosophical Transactions*.^55^ Presenting scientific accounts as matters of fact could make them real, turning the reader into the viewer. In this sense, the travel journals of Lanoy and Goodyear were also of great importance for this purpose. They said almost nothing of the city itself, ‘from which little knowledge could be obtained’,^56^ but they presented a detailed narrative of both travels, to and from Palmyra, therefore completing the picture of the events that took place in the summer of 1678 and the fall of 1691. The authors described the environments that they passed through to get to the city, including local landmarks and natural surroundings, ancient inscriptions, local people and villages, churches and aqueducts, castles and ruins, flora and fauna. All this gave a unique visual strength to the whole experience, producing a kind of objectivity through subjectivity and using the text itself as visual technology.^57^ The fact that the travellers could only rely on their own senses was further specified at the end of Halifax's narrative:

The Reverend and Learned Author of this Account, cannot with Justice be censured, if some Minute Particulars of the History of this Place, have escaped his Memory, being obliged to write without recourse to the Books proper for his purpose, which were not to be had in that Country.^58^

The absence of textual material, documentation and previous research immediately eliminated any possible preconceptions, superstitions or bias, making the accounts, no matter how incomplete, all the more relevant. Furthermore, the travellers had themselves to be trustworthy, otherwise the whole account would not prove believable. For this reason, they were presented as ‘Men of more than ordinary Birth and Education’,^59^ insisting on their origin and background as gentlemen. This would immediately introduce their accounts as products of an educated mind without any hidden agendas. Moreover, the travellers were all Britons, acting abroad in the name of their own country. These two features already contributed in building a frame of reliability that was essential for the making of long-distance knowledge. In addition, secular notions of truthfulness and honesty were recognized as essential parts of religious behaviours, which made Christian gentlemen all the more trustworthy. Halifax was not only a Christian but also a clergyman, making his personal narrative even more faithful and intellectually honest. Finally, Halifax, as well as Huntington before him, operated rather independently from institutional authorities and were inspired to make these dangerous journeys by personal interests and genuine curiosity. This contributed greatly in making his report disinterested and therefore authentic to the eyes of the readers. Gentlemanly origins, national backgrounds, religious motivations and intellectual impartiality were all basic components in the picture of a reliable traveller in early modern England.^60^

Individuals such as Halifax were important contributors to the intellectual programme of *Philosophical Transactions* and the journal itself had in the past readily received similar long-distance reports. Travellers were advised to prepare, among other things, ‘Plotts and draughts of prospects of coasts, Promontories, Islands and Ports’, to mark the position of the place they visited, the natural qualities of air, earth, flora and fauna and to report observations about the inhabitants.^61^ Robert Boyle famously contributed to this agenda in several instances, including in *Philosophical Transactions*, where he insisted on the importance of compiling natural histories through the collections of geographical locations, natural characters, social and cultural traditions of ‘a Countrey, *Great or Small*’.^62^ Making useful and original reports of foreign countries was a duty not only for natural philosophers but also for anyone who embarked in expeditions and travels. Francis Bacon himself had already described this practice as ‘a part of education’, inviting the use of diaries and suggesting the observation of, among other things, ‘monuments’, ‘walls and fortifications of cities and towns’ and ‘antiquities and ruins’.^63^ This was also in line with the development of empirical knowledge and first-person experience, one of the pillars of the Royal Society's methodology. As Stuart Piggott argues:

By the end of the seventeenth century the conditions for the scholarly study of antiquities had in fact been created, largely as a result of the application of the nascent scientific disciplines, and the empirical approach of Bacon and Descartes which lay behind them, which one associates essentially with the Royal Society; among its fellows were to be found several of the leading antiquaries of the time.^64^

The young Royal Society was engaged in a variety of projects involving archaeology, ancient history and antiquarianism, especially before the establishment of the Society of Antiquaries in 1717.^65^ Although the extent, motives and centrality of these themes within the research of the Royal Society have been subjected to debate, it is undeniable that the Society contributed greatly to the development and diffusion of a new kind of antiquarianism, which was becoming a distinctive part of scholarly culture.^66^ Whether officially or informally, the study of ancient artefacts, texts and places was indeed part of both the regular meetings of the Society and its publications. Archaeology as a discipline was building its own synthetic methodology made of organized findings, more distinctive analytical rigour, critical insights and comparative approaches.^67^

In parallel with this, a major contribution towards the development of antiquarianism came from Oxford. The circle of orientalists that developed there was crucial in fostering erudition around Arabic and Hebrew cultures. The University of Oxford thrived throughout the seventeenth century as a major centre of oriental studies. Gibbon described this scholarly culture as ‘the pride of Oxford’,^68^ and both Halifax and Huntington came from precisely this culture. As already mentioned, Halifax was a fellow of Corpus Christi College and there spent almost 20 years between 1670 and 1695. Huntington was a graduate of Merton College and a major contributor to the Bodleian Library, to which he donated many of the findings and documents he brought back from Syria. Belonging to the same generation as Huntington were Halifax's contacts when he was in Aleppo: Bernard and Smith. Dr Edward Bernard became a fellow of St John's College in 1658. He was an extremely erudite arabist and was both a prolific figure within the Oxford circle of orientalists and a qualified mathematician—he succeeded Sir Christopher Wren as Salivian Professor of Astronomy at Oxford in 1673 and was elected FRS in the same year.^69^ Thomas Smith was a graduate of Magdalen College and a passionate orientalist and was elected FRS in 1677. He was in Constantinople between 1668 and 1671 as Chaplain of the Levant Company. Smith went back to London in 1671 and his reports on Constantinople were later published in *Philosophical Transactions.*^70^ Huntington, Bernard and Smith were all acquaintances and were in close contact with other Oxford orientalists and antiquaries such as Edward Pococke, who made invaluable contributions to the study of near-eastern cultures.^71^

The British Levant Company itself was a crucial element for the establishment of stable scholarly channels of communication between Britain and the Orient. Members of the company had been conducting investigations into the historical architecture of the Middle East since the 1660s.^72^ As a result of its secular existence and its relative stability at the time, scholars were able to make peaceful contact with the Arabic-speaking world, and systems of long-distance research could be established and maintained in countries such as Syria and Turkey. The productivity and prosperity of the Levant Company during the second half of the seventeenth century was surely a major component for the intellectual thriving of Arabic and Hebrew studies that existed at Oxford, and it certainly made an impact on the Royal Society as well.^73^ In this sense, Halifax and his company were an informal but important appendix of the Royal Society, and their reports on Palmyra contributed greatly to the accomplishment of its agenda.

## Illustrating the city

The illustration presented with the travel diaries already had a pivotal role in informing the public about the city and in involving the reader in its discovery. As mentioned above, this engraving was published in issue 218 of *Philosophical Transactions*, together with the diaries of Lanoy and Goodyear, who brought and showed to the Society the ‘large and Curious design in paint of the famous ruins of Palmyra’ in October 1695 and ‘another Copy of the Ruins of Palmyra’ in the following month.^74^ The picture was copied from a sketch made on site by a member of the expedition. This might have been G. Hofstede Van Essen, a Dutch painter who, while still in Syria, made a much bigger painting, more than 4 metres long and almost 1 metre high, depicting the ruins of Palmyra.^75^ This painting, dated 1693, was sent from Aleppo to Amsterdam, to the historian and politician Gisbert Cuper.^76^ The engraving published in *Philosophical Transactions* and the painting probably came from the same master sketch. For this reason it has been assumed that Hofstede was part of the 1691 expedition, and that he was in fact the author of both the engraving and the painting.^77^ Despite it being quite a crude depiction of the city, not without certain proportional inaccuracies, the engraving published in *Philosophical Transactions* still proposes a unique and useful view of a late seventeenth-century archaeological site. Moreover, the drawing follows the path that the explorers took during their visit to the city, also making use of ‘visual checkpoints’, very similar to those in Halifax's text: from the far left, at *The Temple Now Inhabited* (the Temple of Bel), all the way through the *Porphyry Pillars* and *The Portico* (the Colonnade Street), to the far right, where we find the *Little Temple* (the Temple of Baal-Shamin), *The Sepulchres* and *The Castle* on top of the hill. By looking at the drawing one can then geographically trace the itinerary of the company and relate Halifax's written descriptions to the graphical representation of the city. These two elements certainly worked together in addressing the reader in a very engaging way, stressing the material substance and features of Palmyra.

The drawing published in *Philosophical Transactions* was not the only one produced by the company during their stay in Palmyra. On 20 November 1695 ‘Mr. Lanoy produced to the Society … the additional draught of a Temple therein and of one of the funeral Monuments there, for which he had the thanks of the Society’.^78^ Of these three drawings only one was published; the other two are now lost. It is difficult to determine to which buildings these drawings were referring. However, if we consider the relationship that seems to have existed between the illustrations and Halifax's text, we can assume that the ‘Temple therein’ referred to the Temple of Bel—or possibly that of Baal-Shamin—given the extent of their written descriptions. As for the ‘one of the funeral Monuments’, this might have been a particular tomb, to which Halifax dedicated a whole paragraph of his report.^79^

The surviving illustration still has a crucial role in informing the public about the rediscovered city. As mentioned in the previous section, the intention of these kinds of report was the establishment of an authority and the codification of standards and values for the transmission of original and distant information. Images displayed and discussed at the Royal Society were treated as an integral part of the knowledge that was produced by textual evidence. The process that led from the production of an image to its dissemination through the system of knowledge-making at the Society expressed a collective intention to authorize and consolidate that knowledge.^80^ In this sense, the original drawings displayed at the Royal Society had as essential a role as the engraving published in *Philosophical Transactions* in establishing the authority of the whole account.

It should also be noted at this point that the late seventeenth century saw the rise of iconographies as primary references for both the production of early modern scientific knowledge and the interpretation of historical evidence.^81^ The knowledge that came back from Palmyra in 1691 might not have been consistent or complete, but it gave concreteness and realism to what would have otherwise remained a mythical, legendary and unreachable city. Through both writing and drawing, Halifax, together with Lanoy and Goodyear, were able to picture publicly the city of Palmyra for the first time in history, contributing to the development of a mode of communication and representation that was still very much in the making. With their personal and passionate documents these travellers were able to produce authentic—and therefore authoritative—knowledge.

## Recording the city

Halifax's approach had the crucial goal of establishing authority and veracity through the accumulation of matters of fact and circumstantial information about Palmyra. In addition to rich descriptions, not without ironic comments and personal notes, the company also attentively collected both inscriptions and measurements.

**Figure 2.**
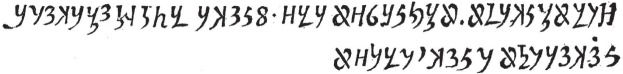
Inscription in the Palmyrene alphabet. (From W. Halifax, ‘A Relation of a Voyage from *Aleppo* to *Palmyra* in *Syria* …’, *Phil. Trans. R. Soc. Lond.* **217**, 83–110 (1695), between pp. 88 and 89.)

Copies of inscriptions bore with them crucial significance. Transcribing ancient writings, translating them and relating them to contemporary religious, social and political knowledge was the most effective and reliable way of bringing ancient facts to the public attention. Such evidence taken from columns and walls occupy the greatest part of Halifax's account. In this sense, his approach was essentially that of an early modern epigraphist. Both in his relation and in the travel diaries of Lanoy and Goodyear, entire pages were dedicated to transcribing almost two dozen ancient inscriptions, analysing the language and deducing facts about the history of Palmyra. Most of these inscriptions were in either Latin or Greek, but Halifax also reported one that was in a language still unknown. This was the Palmyrene language, a Semitic alphabet used in Palmyra during Hellenic and Roman times (figure 2). The existence of this alphabet had already been determined earlier in the century, when Jan Gruter published an example of it in 1616.^82^ However, this time Halifax also understood the key to its decipherment: the ‘[unknown] Character’, he writes, ‘it being added almost under every *Greek* Inscription we saw, and rarely found alone, I am apt to believe it the Native language and Character of the place, and the Matter it contains, nothing else but what we have in the *Greek*’.^83^ This was a crucial discovery, and it actively contributed to the complete decipherment of Palmyrene in 1754. This was the first dead language to be uncovered through inscriptions.^84^

For Halifax, the practice of recording inscriptions was indeed a powerful way to produce accountable information. Inscriptions were generally ‘put up in memory of some, who had behaved themselves … with commendation’ but other historical facts could also be directly observed on these writings. For instance, Palmyra was ‘a Free State, governed by a Senate and People’ and ‘that they were Idolaters is plain by the mention of their Country Gods’.^85^

The epigraphic tradition towards inscriptions had its roots in Renaissance scholarship. Sixteenth-century scholars were already trying to establish this primary material as historical evidence, testing its reliability and questioning its reproducibility.^86^ By transcribing ancient inscriptions, the explorers were quoting the city itself, giving Palmyra its own voice. But this attitude was already changing during the seventeenth century as antiquarianism was gaining its own disciplinary recognition. Scholars such as John Aubrey established a new kind of investigation, made of comparative analysis of material sources, observed for their contextual values. As Alain Schnapp suggests, through the second half of the seventeenth century objects had gained a substantial importance over textual sources as essential material evidence for the uncovering and understanding of historical facts.^87^ The Royal Society was at the very centre of this change towards a more empirical and experimental methodology.^88^ In this sense, Halifax's approach was very much rooted in the secular tradition of epigraphic scholarship and it might even have been considered slightly out of place in *Philosophical Transactions*. As the Secretary of the Society and editor of the journal, Hans Sloane, commented when concluding the reports on Palmyra:

The *Philosophical Reader* is desired to excuse our breaking-in upon the Subject of these *Tracts*, by intermixing *Historical* and *Philological* Matters, as also our exceeding the bounds of an *Extract*: but we hope the Curiosity of the Subject, joyned to the Desires of the *Royal Society*, may make an easie apology suffice.^89^

Be this as it may, the ‘*Historical* and *Philological* Matters’ reported by Halifax and his company still had a crucial role in establishing their exploration of Palmyra as a matter of fact. The other way that the company could report factual information was through measurements. This, however, was more problematic. Given the size and extent of the ruins, the quantity of data necessary for reproducing the buildings with some level of completeness was beyond their capacity and, most probably, their intentions. Nevertheless, Halifax's relation featured rich numeric information. These included the size of the Temenos of the Temple of Bel, ‘a square of 220 yards each side’; the height of a pillar still standing around the site, ‘consisting of seven large Stones’, ‘50 foot' tall, ‘12 Foot and a half' wide; the length and width of the colonnade street, ‘938 Yards … and 40 foot in breadth’,^90^ and several dozens more. To this end, we know that the company had with them a few surveying instruments. When measuring the height of a pillar, Halifax mentioned a ‘quadrant’.^91^ This was a tablet representing a quarter of a circle and could be used to measure the height of the stars during navigation. The quadrant could also be used efficiently to measure the height of a wall or column through the angular ratio between their base and their summit.

Today we know that only some of the numbers mentioned by Halifax were accurate. Moreover, from the way in which they were presented in the text, it would have been extremely difficult at the time to use this short survey to reproduce the buildings. Several of them are still very difficult to place, understand and contextualize today. These measurements, rather than an attempt at a rigid survey, seemed essentially instrumental and worked towards a perceptive and authorial relation of the city. Not unlike the epigraphist's approach towards inscriptions, the surveyor's approach towards measurements was adopted here to bring realism, factuality and therefore accountability to the whole story. On the one hand, fragments of historical facts were recorded from the few inscriptions attentively copied in the reports; on the other, recorded measures gave impressions and glimpses of the grandeur and scale of the city. As these were the only reproducible results available to be transcribed and transported from Palmyra to London, the importance of these words and numbers, although inevitably incomplete, was crucial.

All this mirrors the Royal Society's attitude towards the study of nature and the collection of knowledge from around the world. The descriptive and circumstantial character of these reports was focused primarily on the observable. The Royal Society therefore welcomed these extensive reports from Palmyra with an appreciation that was led by their interests in exactness and precision.

## Publishing the city

William Halifax's letter was sent to Bernard and ‘communicated’ to Smith on 9 October 1695. As for the travel diaries of Lanoy and Goodyear and the drawings, they were personally presented by the authors to the Royal Society a few weeks later. Before being published in *Philosophical Transactions*, they were read for collective approval on 23 October, 20 November and 27 November 1695, as normal procedure dictated.^92^ As mentioned above, the Secretary of the Society and editor of *Philosophical Transactions* was Hans Sloane, a physician and collector of natural curiosities whose assortment of specimens would later be one of the cornerstones of the British Museum.^93^ Sloane was himself a passionate traveller, having spent several months in Jamaica in 1687, after which he published a rich two-volume book on the nature of the islands. When commenting on the accounts on Palmyra, he announced that ‘There may be many other Instructive Remarks made thereon, which still deserve the Consideration of the Learned, and from such the Publick may yet expect a further Account.’^94^

Indeed, after the publication of the Relation and Travel Journals, several other studies on the ancient city were soon proposed to the public attention, starting from *Philosophical Transactions*. Edmund Halley famously dedicated a whole article to the history of Palmyra.^95^ His account was read as a manuscript at the Royal Society on 8 January 1696 and published right next to the Travel Journals in issue 218.^96^ Halley's 15-page article gave a concise history of the city, a short study of its geographical position and ‘some few Remarks’ on the inscriptions found by Halifax's company. These last remarks became a highly valuable source for the decipherment of the Palmyrene language during the following century, making Halley a crucial reference for this particular matter.^97^ At the end of his essay, Halley also asked ‘any curious Traveller, or Merchant residing there’ to establish the Longitudes of Aleppo and other cities nearby—wrongly calculated by Kepler in his *Rudolphine Tables* in 1627—so that he ‘could then pronounce in what Proportion the *Moon*'s Motion does accelerate’. He finally recommended ‘to all that are curious of such matters, to endeavour to get some good Observation made at this Place, to determin the Height of the *Pole* there, thereby to decide the Controversie, whether there hath really been any Change in the *Axis* of the Earth’. Halley had an ongoing interest in mapping and surveying as well as in astronomy. He was also working on a project of mapping cities and regions of the Roman world on a large scale, using astronomical methods. His idea was in fact that of using historical data to inform modern natural philosophy, giving to ancient texts an everlasting and prominent role in shaping scientific knowledge.^98^ Halley's geographical projects further suggest the permeability of antiquarian studies to other early modern scientific disciplines.

All the documents produced on Palmyra—Halifax's letter, the diaries, the drawing and Halley's paper—were printed together in the two consecutive issues of *Philosophical Transactions*. Interestingly, the earliest news of the discovery of Palmyra arrived in France and was briefly mentioned in *Journal des Sçavans* as early as June 1692. A few English gentlemen were said to have seen 400 marble or porphyry columns, temples still intact, tombs and Greek and Latin inscriptions.^99^ Moreover, the travel reports of Halifax, Lanoy and Goodyear were not the only copies that were produced. Coenraad Calckberner, the Dutch Consul in Aleppo, had sent a copy of the documents to Gisbert Cuper in July 1692, together with coins, other artefacts and a painting depicting the ruins of Palmyra.^100^ Cuper's intention was to publish a complete account of the travel, together with a commentary, after having translated the whole thing from English. In fact, Cuper was in possession of additional information and original material. Thomas Smith himself was aware of his project: as he announced in *Philosophical Transactions*, the accounts published there were meant to be nothing more than a pleasant ‘appetizer’ (πρόποµ*α*), until Cuper's bigger oeuvre would be published.^101^ Because this never happened, the relations in *Philosophical Transactions* remain the first published accounts of this journey to Palmyra.

After having resigned his chaplaincy in Aleppo in November 1695, William Halifax arrived back in England in early 1696.^102^ On his way home he stopped in Rome, looking for a Palmyrene inscription that had been copied by Jacob Spon in his *Miscellanea*, a collection of various antiquarian studies published in 1674. He was unable to find the original, but he was helped by Octavian Pulleyn, former printer to the Royal Society during the 1660s, who joined the search in March 1696. Pulleyn then sent a letter to London with a copy of the inscription he found in Rome. This letter, reporting the earlier ‘fruitless’ enquiry as well as the successful one, was published in *Philosophical Transactions* in May 1697, together with drawings of the Palmyrene and other Etruscan inscriptions.^103^

Publications of Halifax's account on Palmyra also continued during the eighteenth century. In 1774 the archaeologist Thomas Kerrich found in Rome a manuscript copy of Halifax's relation—possibly left by him during his journey. The manuscript was then given to Albert Hartshorne and published in 1890 in *Palestine Exploration Quarterly*. This transcription included a description of the journey to and from Palmyra. The description itself differs slightly from that published in *Philosophical Transactions* but most notably it included several more inscriptions in both Greek and Palmyrene.^104^

Indeed, the archaeological legacy of Palmyra started in *Philosophical Transactions* and had a powerful echo throughout the following centuries, fostering further research and archaeological discoveries. It is probably fair to say that the accounts published in *Philosophical Transactions* became a symbol for the rediscovered city. For instance, the drawing was copied and reprinted in a number of travel books, often—but not necessarily—together with the entire account.^105^ One of the first examples of this process was Abednego Seller's *The Antiquities of Palmyra*, published in 1696, presented to the Royal Society on 14 October^106^ and reviewed in issue 223 of *Philosophical Transactions* later that same year. The book dealt extensively with the cultural, political and social history of the city and featured some observations on the inscriptions found by Halifax and already commented upon by Halley. Seller's book also included a reproduction of the large engraving. A few years later, in 1698, Bernard, Smith and Huntington—all directly involved in the making and dissemination of the first discovery of the city—published a short pamphlet on the inscriptions found by the company in Palmyra. Their book, called *Inscriptiones Græcæ Palmyrenorum*, featured all of the 23 inscriptions published in *Philosophical Transactions*, translated into Latin and commented upon by the three authors.^107^

From the turn of the century, Palmyra became more and more present in travel accounts, architectural publications and archaeological studies: from Le Bruyn's *Voyage au Levant* (1698)^108^ to Fischer von Erlach's renowned *Plan of Civil and Historical Architecture*, first published in 1721,^109^ to Robert Wood's celebrated *The Ruines of Palmyra* (1753).^110^ At the beginning of his book, Wood did not hide his debt to his older compatriots, who inspired the expedition and ‘wrote with so much candour and regard to truth’ an account that was ‘the only one I have even seen of this place’. Halifax's account of Palmyra was therefore considered to be a truly unique achievement. With Wood's *Ruines* the city became the symbol of the British conquest of the neoclassical architectural language.^111^

## Conclusion

When William Halifax decided to reach the city of Palmyra—as did Huntington before him—his project was intended as a rather independent venture and it is likely that the very idea of this journey was essentially autonomous. *Philosophical Transactions* then actively worked in producing, materializing and promoting Palmyra's rediscovered remains, reallocating the value of those independent travels into a collective realm. In this process the Royal Society and its contacts in Oxford served as the key link between London and the Orient. The study of Palmyra was publicly presented as a scientific and philosophical matter, involving astronomers, philosophers, epigraphists, historians and classicists. The journey then became a large-scale effort that could even go beyond antiquarian scholarship—as proved by Halley's involvement. This process of intellectual rescaling and international scholarly dissemination needs to be regarded as one of the most influential outcomes of *Philosophical Transactions* in its early years.

*Philosophical Transactions* has already been recognized as a crucial editorial undertaking within both the history of communications and the development of the early Royal Society.^112^ The first few decades of the journal's life were, however, highly uncertain, both economically and institutionally.^113^ Oldenburg ventured this enterprise largely on a personal basis and for decades the Royal Society itself did not consider the journal its own editorial responsibility. *Philosophical Transactions* underwent progressive stabilization mainly as a result of the changing experimental programme at the Society and the influence and contribution of its several editors.^114^ This initial flexibility and precariousness suggests that the reports of Palmyra, given the extensive space they occupied in *Philosophical Transactions*—to the point of needing an apology from the editor—had the potential to contribute to the validation of the authority of the journal itself. It is not unlikely that the intellectual image of *Philosophical Transactions* could benefit from the success of these reports and from their continental dimension, as proved by the several references to the journal made by later scholars in their successive works on Palmyra.

Moreover, these reports could actively shift the Society's own programme in a certain direction. As has been pointed out, research made at the Royal Society was often directly influenced by the accounts, specimens and curiosities brought as a result of long-distance journeys.^115^ Travels such as those to Palmyra could then direct the interests of scholars in and around the Royal Society, fostering the Society's agenda through their antiquarian research on Palmyra.^116^ The rediscovery of Palmyra and its public appearance in the 1690s therefore also need to be included among those researches that moved *Philosophical Transactions*, its readership and the Royal Society as a whole towards the institutional validation of antiquarian knowledge.

Funding: This work was supported by the Arts and Humanities Research Council.

## Notes

1 In about 20 May 2015 troops belonging to the so-called Islamic State reached Tadmor and the ancient ruins of Palmyra. From late August to October 2015, news of the destruction of several parts of the city—including the Temple of Baal-Shamin, the inner Temple of Bel, the Monumental Arch and several Tower Tombs—has come to the attention of the public. Statues from Palmyra's Archaeological Museum were destroyed and other archaeological artefacts were looted. Various parts of the ancient city, such as the Roman Theatre, have been used to stage executions of soldiers as well as prisoners and civilians, including Palmyra's former director of antiquities, Khaled al-Assad.

2 See in particular S. Shapin, ‘Pump and circumstance: Robert Boyle's literary technology’, *Social Stud. Sci.* **14**, 481–520 (1984); *idem*, ‘The house of experiment in seventeenth-century England’, *Isis* **79**, 373–404 (1988); *idem*, *A social history of truth. Civility and science in seventeenth-century England* (University of Chicago Press, 1994). The first two of these were reprinted in *idem*, *Never pure: historical studies of science as if it was produced by people with bodies, situtated in time, space, culture, and society, and struggling for credibility and authority* (Johns Hopkins University Press, Baltimore, MD, 2010), Part II (‘Places and Practices’), pp. 57–116.

3 Shapin, ‘The house of experiment’, *op. cit*. (note 2), p. 375.

4 M. Feingold, ‘Oriental studies’, in *The history of the University of Oxford* (ed. N. Tyacke), vol. 4 (*Seventeenth-century Oxford*), pp. 449–503 (Oxford Scholarship Online, 2011).

5 See, for example, A. Schnapp, *The discovery of the past* (British Museum Press, London, 1996), esp. ch. 3: M. C. W. Hunter, ‘The Royal Society and the origins of British archaeology (parts I and II)’, *Antiquity* **45**, 113–121 and 187–192 (1971), reprinted in M. C. W. Hunter, *Science and the shape of orthodoxy* (Boydell Press, Woodbridge, 1995); G. Parry, *The trophies of time. English antiquaries of the seventeenth century* (Oxford University Press, 1995).

6 H. Oldenburg, ‘A preface to this Seventh Year of these Transactions’, *Phil. Trans*. **6**, 2087–2093 (1671), at p. 2089.

7 See, for instance, M. Lister, ‘An Account of a Roman Monument …’, *Phil. Trans*. **13**, 70–74 (1683); ‘Some Observations upon the Ruins of a Roman Wall …’, *Phil. Trans*. **13**, 238–242 (1683); ‘A Letter … with Two Draughts of the Famous Persepolis’, *Phil. Trans*. **18**, 117–118 (1694); R. Thoresby, ‘A Letter … Giving an Account of a Roman Pottery …’, *Phil. Trans*. **19**, 319–320 (1695); ‘Part of a Letter … Concerning a Roman Shield’, *Phil. Trans*. **20**, 205–208 (1698); ‘Part of a Letter … Concerning Some Roman Coins …’, *Phil. Trans*. **23**, 1156–1158 (1702); J. Lyster, ‘A Description of a Roman Sudatory …’, *Phil. Trans*. **25**, 2226–2227 (1706); T. Smith, ‘Historical Observations Relating to Constantinople …’, *Phil. Trans*. **13**, 335–346 (1683); ‘An Account of the City of Prusa … and a Continuation of the Historical Observations Relating to Constantinople …’, *Phil. Trans*. **14**, 431–454 (1684).

8 See, for instance, ‘Directions for Sea-Men, Bound for Far Voyages’ *Phil. Trans.* **1**, 140–143 (1665); ‘An Appendix to the Directions for Seamen, Bound for Far Voyages’ *Phil. Trans.* **1**, 147–149 (1666); R. Boyle, ‘General Heads for a Natural History of a Countrey, Great or Small, Imparted Likewise by Mr. Boyle’, *Phil. Trans.* **1**, 186–189 (1666).

9 D. Carey, ‘Compiling nature's history: travellers and travel narratives in the early Royal Society’, *Ann. Sci.* **54**, 269–292 (1997); M. Hunter, ‘Robert Boyle and the early Royal Society: reciprocal exchange in the making of Baconian science’, *Br. J. Hist. Sci.* **40**, 1–23 (2007).

10 The Levant Company had existed for more than a century and had stations all around the Mediterranean coasts of the Middle East, managing commerce with the Orient. See A. C. Wood, *A history of the Levant Company* (F. Cass, London, 1935). For a colourful account of the life of these men in Aleppo, see H. Teonge, *The Diary of Henry Teonge, anno 1675–1679* (R. Bentley, London, 1825).

11 S. Mills, ‘The English chaplains at Aleppo: exploration and scholarship between England and the Ottoman Empire, 1620–1760’, *Bull. Coun. Br. Res. Levant* **6**, 13–20 (2011). For more on Robert Huntington's life and manuscripts, see his biography by Thomas Smith, *D. Robert Huntingtoni … Epistolæ* (London, 1704). See also Feingold, *op. cit*. (note 4), pp. 491–492; A. Hamilton, ‘Robert Huntington’, in *Oxford dictionary of national biography*, vol. 28, pp. 938–940 (Oxford University Press, 2004; online edn, 2008, http://www.oxforddnb.com/view/article/14242 (requires subscription)). Interestingly, Huntington's only publication on oriental antiquities was an article on the Porphyry Pillars in Egypt, which appeared as R.H., ‘A Letter from Dublin to the Publisher of these Tracts, concerning the Porphyry Pillars in Egypt’, *Phil. Trans*. **14**, 624–629 (1684).

12 W. Halifax, ‘A Relation of a Voyage from *Aleppo* to *Palmyra* in *Syria*; Sent by the Reverend Mr. *William Halifax* to Dr. *Edw. Bernard* (Late) *Savilian* Professor of Astronomy in *Oxford*, and by Him Communicated to Dr. *Thomas Smith. Reg. Soc. S.*’, *Phil. Trans. R. Soc. Lond.* **217**, 83–110 (1695). Bernard, Smith and Huntington were lifelong colleagues and friends, and they were all key figures within the collection and dissemination of Halifax's reports on Palmyra in subsequent years (see below).

13 Halifax was also the author in 1685 of an English translation of Deschales's Euclid, published in French in the previous year. See J. B. Pearson, *Biographical Sketches of the Chaplains to the Levant Company, 1611–1706* (Cambridge, 1883), p. 24; D. Gray, ‘Hallifax, William (1655–1721/2)’, in *Oxford dictionary of national biography*, vol. 25, pp. 705–706 (Oxford University Press, 2004; online edn, 2008, http://www.oxforddnb.com/view/article/12018 (requires subscription)).

14 Factors had to undertake a period of seven years of apprenticeship—three in London and four in the Levant—after which they would become free traders and had the company's permission to act as factors for the merchants in London. Members of the Levant Company could act independently, although they often chose to work in partnerships. Wood, *op. cit*. (note 10), pp. 215–216.

15 Teonge, *op. cit*. (note 10), p. 160.

16 National Archives: SP 100/73, Part 1(2) and Part 1(3). Lanoy and Goodyear appear regularly in the meetings of the Levant Company throughout the 1670s and 1680s.

17 ‘An extract of the Journals of Two Several Voyages of the English Merchants of the Factory of Aleppo, to Tadmor, Anciently Call'd Palmyra’, *Phil. Trans.* **19**, 129–160 (1695).

18 ‘A view of the Ruines of Palmyra alias Tadmor, taken on the Southern Side’, *Phil. Trans. R. Soc. Lond.* **218**, 83–110 (1695).

19 Halifax, *op. cit*. (note 12), pp. 84–85.

20 *Ibid.*, pp. 87–88.

21 *Ibid.*, p. 88.

22 *Ibid.*, pp. 90–91.

23 *Ibid.*, p. 86. This village in the ruins remained inhabited for several centuries, being forcedly dismantled by the French government in the late 1920s, when Palmyra was thoroughly excavated and turned into the archaeological site we know today.

24 *Ibid.*, p. 91.

25 I. Browning, *Palmyra* (Chatto & Windus, London, 1979), fig. 18, p. 122.

26 Halifax noted that these pillars were made ‘of one entire stone’. This is quite a unique feature that distinguishes the portico of Diocletian's Baths from most buildings in Palmyra. See also Browning, *op. cit*. (note 25), p. 60.

27 The ‘several Openings for Gates’ that they observed along the western side of the ‘Piazza’ are probably the archways to the Transverse Streets and the entrances to the stage of the Theatre. In addition, the main doorway that led into the building was in fact adorned with four columns, which just as Halifax described were ‘not standing in a Line with the others of the Wall, but placed by couples in Front of the Gate’. Halifax, *op. cit*. (note 12), pp. 101–102.

28 Ibid.

29 *Ibid.*, p. 104.

30 Ibid.

31 *Ibid.* See J. B. Ward-Perkins, *Roman imperial architecture* (Yale University Press, London, 1981), p. 360.

32 Halifax, *op. cit*. (note 12), pp. 105–107.

33 ‘An extract …’, *op. cit.* (note 17), pp. 146–147.

34 See, for example, L. Soo, ‘Reconstructing antiquity. Wren and his circle and the study of natural history, antiquarianism and architecture at the Royal Society’, PhD thesis, Princeton University (1989), p. 146.

35 The report did not include two other sections of the Colonnade Street, the Funerary Temple and Diocletian's Camp, all part of Palmyra's ancient history and dating from the first and second centuries.

36 As I discuss below, the group might have included a Dutch painter called Hofstede van Essen.

37 See, for example, W. E. Houghton Jr, ‘The English virtuoso in the seventeenth century (parts 1 and 2)’, *J. Hist. Ideas* **3**, 51–73 and 190–219 (1942); S. Bann, *Under the sign. John Bargrave as collector, traveler, and witness* (University of Michigan Press, Ann Arbor, MI, 1994); Parry, *op. cit*. (note 5); M. C. Hunter, *Wicked intelligence. Visual art and the science of experiment in Restoration London* (University of Chicago Press, 2013).

38 Soo, *op. cit*. (note 34), p. 148.

39 The authors of the travel diaries also ironically noted that ‘this Notion sticks in the Heads of these people, That the *Franks* go to see old Ruines, only because they there meet with Inscriptions which direct them to some hid Treasures’. ‘An extract …’, *op. cit*. (note 17), p. 155.

40 Wood, *op. cit*. (note 10), pp. 223–224.

41 Halifax, *op. cit*. (note 12), p. 96.

42 M. Zuchowska, ‘Palmyra and the far eastern trade’, *Palmyrene Stud.* **12**, 381–387 (2013).

43 T. M. Clarke, ‘The discovery of Palmyra’, *Architect. Rev.* **101**, 89–96 (1947), at p. 89.

44 See, for instance, M. Hunter, ‘A “college” for the Royal Society: the abortive plans of 1667–1668’, *Notes Rec. R. Soc*. **38**, 159–186 (1984), p. 160.

45 Halifax, *op. cit*. (note 12), p. 91.

46 *Ibid.*, p. 89.

47 *Ibid.*, p. 90.

48 *Ibid.*, p. 95.

49 *Ibid.*, p. 91.

50 *Ibid.*, pp. 92, 87.

51 *Ibid.*, p. 102.

52 ‘As soon as you are entred within the Court, you see the remainders of two Rows of very Noble Marble Pillars … Advancing then towards the North, you have before you a very tall and stately Obelisk or Pillar …. On each hand of this, … you see two other large Pillars … Proceeding forward, … you come to a Magnificent Entrance’. Halifax, *op. cit*. (note 12), pp. 89, 92 and 95.

53 Shapin, *A social history of truth*, *op. cit*. (note 2), pp. 243–247; *idem*, ‘Pump and circumstance’, *op. cit*. (note 2).

54 Shapin, ‘Pump and circumstance’, *op. cit*. (note 2), pp. 490–491.

55 See Shapin, ‘The house of experiment’ and ‘Pump and circumstance’, *opp. cit*. (note 2); P. Dear, ‘Totius in Verba: rhetoric and authority in the Early Royal Society’, *Isis* **76**, 144–161 (1985); A. G. Gross, Joseph E. Harmon and Michael S. Reidy, *Communicating science: the scientific article from the* 17th *century to the present* (Oxford University Press, 2002), esp. ch. 2; A. Hultzsch, *Architecture, travellers and writers. Constructing histories of perception 1640–1950* (Legenda, Oxford, 2014), pp. 44–45.

56 ‘An extract …’, *op. cit.* (note 17), p. 146.

57 Hultzsch, *op. cit*. (note 55), p. 35; Shapin, ‘Pump and circumstance’, *op. cit*. (note 2), p. 491.

58 Halifax, *op. cit*. (note 12), p. 110.

59 ‘An extract …’, *op. cit*. (note 17), p. 129.

60 Shapin, *A social history of truth*, *op. cit*. (note 2), pp. 74–86. It should be noted that, whereas gentlemanly origins and religious backgrounds were considered favourable indicators of truth-telling, such veracity was hardly verifiable. Shapin makes this point quite clear, stressing that the social and normative frameworks of trust as well as its practical realm were, in fact, parts of a complex system of values and notions that was never unilaterally solved. See Shapin, *A social history of truth*, *op. cit*. (note 2), esp. chs 5 and 6.

61 ‘Directions for Sea-Men’, *op. cit*. (note 8), p. 142.

62 Boyle, *op. cit*. (note 8). See Carey, *op. cit*. (note 9), pp. 272–274.

63 F. Bacon, ‘Of Travel (essay 18)’, in *Essayes or Counsels, Civill and Morall* (London, 1625), p. 100.

64 S. Piggott, *Ruins in a landscape*. *Essays in antiquarianism* (Edinburgh University Press, 1976), p. 20.

65 See, for example, Hunter, *op. cit.* (note 5), pp. 181–200; M. Walker, *Francis Vernon, the early Royal Society and the first English encounter with Greek architecture*, University of Oxford, History of Art Department's Research Seminar, 20 February 2013 (see https://podcasts.ox.ac.uk/research-seminar-francis-vernon-early-royal-society-and-first-english-encounter-greek).

66 See Hunter, *op. cit*. (note 5); Schnapp, *op. cit*. (note 5).

67 See, for example, M. C. W. Hunter, *John Aubrey and the realm of learning* (Duckworth, London, 1975).

68 Feingold, *op. cit*. (note 4), p. 449.

69 Thomas Smith wrote a biographical sketch of Bernard in his *D. Robert Huntingtoni …*, *op. cit*. (note 11). For more, see H. de Quehen, ‘Bernard, Edward (1638–1697)’, in *Oxford dictionary of national biography*, vol. 5, pp. 424–426 (Oxford University Press, 2004; online edn, 2009, http://www.oxforddnb.com/view/article/2240 (requires subscription)).

70 L. Soo,‘The English in the Levant: social networks and the study of architecture’, in *‘The mirror of Great Britain’. National identity in seventeenth-century British architecture*, (ed. O. H. Turner), pp. 209 231 (Spire Books, Reading, 2012). See also T. Harmsen, ‘Smith, Thomas (1638–1710)’, in *Oxford dictionary of national biography*, vol. 51, pp. 335–337 (Oxford University Press, 2004, http://www.oxforddnb.com/view/article/25912 (requires subscription)). For more on Smith and the Oxford circle, also touching on the reports on Palmyra, see the Smith MS collections at the Bodleian Library (esp. MS Smith 5, 45, 46 and 102).

71 G. J. Toomer, *Eastern wisdom and learning: the study of Arabic in seventeenth-century England* (Oxford University Press, 1996), esp. chs 6–9; P. M. Holt, *Studies in the history of the Near East* (Frank Cass, London, 1973), esp. ch. 1.

72 Soo, *op. cit*. (note 70), pp. 209–231.

73 See G. A. Russell, ‘The seventeenth century, the age of the “Arabick”’, in *The ‘Arabick’ interest of the natural philosophers in seventeenth-century England* (ed. G. A. Russell), pp. 1–19 (Brill, Leiden, 1994).

74 Journal Book of the Royal Society, JBO/9, pp. 199, 202. See also Early Letters, EL/WS/55.

75 The painting is currently held—and used to be hung—in the Library of the University of Amsterdam.

76 B. Chen, ‘Digging for antiquities with diplomats: Gisbert Cuper (1644–1716) and his social capital’, *Republ. Lett. J. Study Knowl. Politics Arts* **1**, 13–15 (2009).

77 Browning, *op. cit*. (note 25), fig. 18, pp. 54–55.

78 JBO/9, p. 202.

79 Halifax, *op. cit*. (note 12), p. 107.

80 See, for instance, S. Kusukawa, ‘Picturing knowledge in the early Royal Society: the examples of Richard Weller and Henry Hunt’, *Notes Rec. R. Soc.* **65**, 273–294 (2011).

81 See, for example, ‘Curiosity drawn: early modern science as a visual pursuit’, *Huntington Lib. Q.* **78 **(spec. iss.), 141–415 (2015); R. Baldasso, ‘The role of visual representation in the scientific revolution: a historiographic enquiry’, *Centaurus* **48**, 69–88 (2006); P. Burke, ‘Images as evidence in seventeenth century Europe’, *J. Hist. Ideas* **64**, 273–296 (2003).

82 J. Gruter, *Inscriptionum Romanarum Corpus Absolutissimum* (Heidelberg, 1616).

83 Halifax, *op. cit.* (note 12), p. 89.

84 For a comprehensive account of this matter, see P. T. Daniels, ‘“Shewing of Hard Sentences and Dissolving of Doubts”: the first decipherment’, *J. Am. Orient. Soc.* **108**, 419–436 (1988).

85 Halifax, *op. cit*. (note 12), pp. 92, 94 and 97.

86 W. Stenhouse, *Reading inscriptions and writing ancient history. Historical scholarship in the Late Renaissance* (Institute of Classical Studies, London, 2005) esp. chs 3 and 5.

87 Schnapp, *op. cit*. (note 5).

88 Hunter, *op. cit*. (note 5), pp. 113–117.

89 H. Sloane, in E. Halley, ‘Some Account of the Ancient State of the City of Palmyra, with Short Remarks upon the Inscriptions Found there’, *Phil. Trans.* **19**, 160–175 (1695), p. 175. See also Soo, *op. cit.* (note 34), pp. 125–126.

90 Halifax, *op. cit*. (note 12), pp. 87, 92 and 95.

91 *Ibid.*, p. 92.

92 JBO/9, pp. 199 and 202–203. A first draft copy of Lanoy and Goodyear's accounts consists of the first four pages of the diary (Classified Papers of the Royal Society, Cl.P/7i/50). A second, complete version of the report was written in the official Register Books (RBO/8/14).

93 See, for example, A. MacGregor (ed.), *Sir Hans Sloane: collector, scientist, antiquary, founding father of the British Museum* (British Museum Press, London, 1994).

94 Sloane, *op. cit.* (note 89).

95 E. Halley, *op. cit.* (note 89).

96 JBO/9, p. 209; Cl.P/21/44.

97 See Daniels, *op. cit*. (note 84), pp. 419–420.

98 N. J. W. Thrower, ‘Edmond Halley as a thematic geo-cartographer’, *Ann. Assoc. Am. Geogrs* **59**, 652–676 (1969). See also A. Cook, ‘Foreign travels’, *Notes Rec. R. Soc. Lond*. **53**, 291–293 (1999).

99 ‘On m'a mandé d'Alep, que des Gentils-hommes Anglois touchez de la curiosité de visiter les ruines de Palmyra, y avoient trouvé quatre cent colonnes de marbre ou de porphire, des temples encore entiers, des tombeaux, des inscriptions Greques & Latines, desquelles j'espere recevoir bientôt des copies’. In ‘Extrait d'Une Lettre de Mr. Cuper, à Mr. l'Abbé Nicaise’, *J. Sçavans* **25**, 298 (1692). A report of this short account was then copied in the following year by John Ray in his *Collection of Curious Travels and Voyages*, printed in London in 1693 (pp. 155–156).

100 Chen, *op. cit.* (note 76).

101 Halifax, *op. cit*. (note 12), pp. 83–84. A Latin translation of the journey appeared in 1697 in the journal *Bibliotheca Librorum Novorum*. See Chen, *op. cit*. (note 76), p. 15.

102 Pearson, *op. cit*. (note 13), p. 32.

103 O. Pulleyn, ‘Part of a Letter from Mr. Octavian Pulleyn, dated, Rome March 16 1696, giving an account of an inscription there found in the Language of the Palmereni; and another in the Etruscan Language found on an old Urne’, *Phil. Trans.* **19**, 537–539 (1695). See also C. A. Rivington, ‘Early printers to the Royal Society 1663–1708’, *Notes Rec. R. Soc. Lond.*. **39**, 1–27 (1984), at pp. 10–11.

104 ‘Relation of a Voyage to Tadmor in 1691’, *Palest. Explor. Q.* **22**, 273–303 (1890). The following article consisted of a study of the Palmyrene inscriptions found by Halifax and their subsequent translations by different authors. See C. R. Corner, ‘Notes on the Voyage to Tadmor’, *Palest. Explor. Q.* **22**, 303–306 (1890).

105 For a chronological account of travels and publications on Palmyra throughout the centuries, see Clarke, *op. cit*. (note 43).

106 JBO/10/1.

107 E. Bernard and T. Smith, *Inscriptiones Græcæ Palmyrenorum* (Francis Halma, Utrecht, 1698).

108 Le Bruyn's book also featured a translation into French of Halifax's entire account from *Philosophical Transactions*, as well as the illustration ‘un peu rendu meilleur’ by the author by adding a few broken columns on the foreground. His compatriot Cornelius Loos was sent shortly afterwards by King Karl XII on another field trip to collect drawings and accounts on ancient rarities and objects from all over the Middle East. Loos's accounts on Palmyra, which are now mostly lost, again feature a survey of city from a traveller's point of view. Loos's remaining drawings are now held at the National Museum in Stockholm. For an account of these documents, see W. Anderson, ‘Cornelius Loos’, in *Stralsund. Tagebl.* **17** (3) (1927).

109 The book was a rich and original collection of existing buildings and imaginary architectures from Europe and Asia, put together with some of the author's own designs. The ruins of Palmyra were featured in the section dedicated to ‘Lesser Known Ancient Roman Buildings’. The chapter dedicated to the city included a short description of its ancient and recent history, a celebration of Halifax and Le Bruyn for their travels, as well as a large drawing depicting a view of the city. The drawing was most probably taken from the one published in *Philosophical Transactions* and included most of the same details with some—quite imaginative—additions in the foreground.

110 See, for example, C. A. Hutton, ‘The travels of “Palmyra” Wood in 1750–1751’, *J. Hellen. Stud.* **47**, 102–128 (1927). Interestingly, the first plate in Wood's book, depicting a large view of Palmyra, is taken from the northeast, a very similar point of view to the drawing made during the 1691 expedition. *The Ruines of Palmyra* was also featured in *Philosophical Transactions* in late 1753, in an article on the 23 inscriptions originally published by Halifax and again by Wood. See J. Swinton, ‘An Explication of All the Inscriptions in the Palmyrene Language and Character Hitherto Publish'd. In Five Letters from the Reverend Mr. John Swinton, M. A. of Christ-Church, Oxford, and FRS to the Reverend Thomas Birch, D.D. Secret. R.S.’, *Phil. Trans.* **48**, 690–756 (1753).

111 The last contribution of the eighteenth century to the city of Palmyra was Louis-François Cassas's *Voyage Pittoresque de la Syrie* (Paris, 1799), demonstrating once again the artistic value of the ruins and bringing the city to the French public.

112 D. Kronick, ‘Notes on the printing history of the early “Philosophical Transactions”’, *Libr. Cult.* **25**, 243–268 (1990); M. Boas Hall, ‘The Royal Society's role in the diffusion of information in the seventeenth century’, *Notes Rec. R. Soc.* **29**, 173–192 (1975); *idem*, ‘Oldenburg and the art of scientific communication’, *Br. J. Hist. Sci.* **2**, 277–290 (1965); E. N. da C. Andrade, ‘The birth and early days of the *Philosophical Transactions*’, *Notes Rec. R. Soc. Lond*. **20**, 9–27 (1965); D. McKie, ‘The origins and foundation of the Royal Society of London’, *Notes Rec. R. Soc. Lond*. **15**, 1–37 (1960).

113 N. Moxham, ‘Fit for print: developing an institutional model of scientific periodical publishing in England, 1665–*ca*. 1714’, *Notes Rec*. **69**, 241–260 (2015); A. Johns, *The nature of the book. Print and knowledge in the making* (University of Chicago Press, 1998), esp. ch. 7.

114 Moxham, *op. cit*. (note 113), pp. 253–255; A. Fyfe, J. McDougall-Waters and N. Moxham, ‘350 years of scientific periodicals’, *Notes Rec*. **69**, 227–239 (2015), at pp. 229–230.

115 See, for example, Carey, *op. cit*. (note 9), esp. pp. 275–279; *idem*, ‘Locke, travel literature, and the natural history of man’, *Seventeenth Cent.* **11**, 159–280 (1996); J. Stoye, *English travellers abroad*, revised edn (Yale University Press, London, 1989).

116 See, for instance, Hunter, *op. cit*. (note 5), p. 115.

